# Menstrual Hygiene Practices, WASH Access and the Risk of Urogenital Infection in Women from Odisha, India

**DOI:** 10.1371/journal.pone.0130777

**Published:** 2015-06-30

**Authors:** Padma Das, Kelly K. Baker, Ambarish Dutta, Tapoja Swain, Sunita Sahoo, Bhabani Sankar Das, Bijay Panda, Arati Nayak, Mary Bara, Bibiana Bilung, Pravas Ranjan Mishra, Pinaki Panigrahi, Sandy Cairncross, Belen Torondel

**Affiliations:** 1 Disease Surveillance Laboratory, Asian Institute of Public health, Bhubaneswar, Odisha, India; 2 Department of Occupational and Environmental Health, University of Iowa, Iowa City, Iowa, United States of America; 3 Department of Obstetrics and gynaecology, Capital Hospital, Bhubaneswar, Odisha, India; 4 Department of Obstetrics and gynaecology, Ispat General Hospital, Rourkela, Odisha, India; 5 Departments of Epidemiology and Pediatrics, Center for Global Health and Development, College of Public Health, University of Nebraska Medical Center, Omaha, Nebraska, United States of America; 6 Department of Disease Control, London School of Hygiene and Tropical Medicine, London, United Kingdom; University of Illinois at Urbana-Champaign, UNITED STATES

## Abstract

Menstrual hygiene management (MHM) practices vary worldwide and depend on the individual’s socioeconomic status, personal preferences, local traditions and beliefs, and access to water and sanitation resources. MHM practices can be particularly unhygienic and inconvenient for girls and women in poorer settings. Little is known about whether unhygienic MHM practices increase a woman’s exposure to urogenital infections, such as bacterial vaginosis (BV) and urinary tract infection (UTI). This study aimed to determine the association of MHM practices with urogenital infections, controlling for environmental drivers. A hospital-based case-control study was conducted on 486 women at Odisha, India. Cases and controls were recruited using a syndromic approach. Vaginal swabs were collected from all the participants and tested for BV status using Amsel’s criteria. Urine samples were cultured to assess UTI status. Socioeconomic status, clinical symptoms and reproductive history, and MHM and water and sanitation practices were obtained by standardised questionnaire. A total of 486 women were recruited to the study, 228 symptomatic cases and 258 asymptomatic controls. Women who used reusable absorbent pads were more likely to have symptoms of urogenital infection (AdjOR=2.3, 95%CI1.5-3.4) or to be diagnosed with at least one urogenital infection (BV or UTI) (AdjOR=2.8, 95%CI1.7-4.5), than women using disposable pads. Increased wealth and space for personal hygiene in the household were protective for BV (AdjOR=0.5, 95%CI0.3-0.9 and AdjOR=0.6, 95%CI0.3-0.9 respectively). Lower education of the participants was the only factor associated with UTI after adjusting for all the confounders (AdjOR=3.1, 95%CI1.2-7.9). Interventions that ensure women have access to private facilities with water for MHM and that educate women about safer, low-cost MHM materials could reduce urogenital disease among women. Further studies of the effects of specific practices for managing hygienically reusable pads and studies to explore other pathogenic reproductive tract infections are needed.

## Introduction

Menstrual hygiene is an important issue that affects healthy adolescent girls and pre-menopausal adult women monthly. Around the world women have developed their own personal strategies to cope with menstruation, which vary from country to country and depend on economic status, the individual’s personal preferences, local traditions and cultural beliefs and education status [1–3]. Often methods of management can be unhygienic and inconvenient, particularly in poorer settings. In India, between 43% and 88% of girls wash and reuse cotton cloths rather than use disposable pads [4,5]. However reusable material may not be well sanitized because cleaning is often done without soap and with unclean water, and social taboos and restrictions force drying indoors, away from sunlight and open air [5]. Unhygienic washing practices are particularly common in rural areas and amongst women and girls in lower socio-economic groups. Menstrual hygiene management (MHM) is also likely to be affected by contextual factors, such as access to places where women can manage menstruation-related washing in privacy and comfort. These factors are influenced by having access to water, hygiene and sanitation facilities at the household, and their link with MHM and with urogenital infections has never been studied in detail.

Poor MHM may increase a woman’s susceptibility to reproductive tract infections (RTI).

A limited body of evidence supports the premise that bacterial vaginosis (BV) may be more common in women with unhygienic menstrual hygiene management (MHM) practices [1,6,7]. Bacterial vaginosis is a poly-microbial syndrome characterized by the imbalance of resident bacterial flora in the vagina. The normal vaginal flora is dominated by hydrogen peroxide producing *lactobacilli*. In BV, there is a reduction in the population of lactobacilli with a simultaneous increase in a diverse community of bacteria including *Gardnerella vaginalis*, *Pretovella sp*, *Bacterioides sp*, *Peptostreptococcus sp*, *Mycoplasma hominis*, *Ureoplasma urea*, *Mobiluncus species (spp)*, and other bacterial *species* [8]. As a girl progresses from puberty into womanhood, RTIs potentially triggered by poor MHM could affect her reproductive health. Studies have shown women with BV may be at higher risk of adverse pregnancy outcomes like preterm birth [9–11], acquisition of sexually transmitted infections [12,13] and development of pelvic inflammatory disease (PID) [14–16].

RTIs are a major public health concern worldwide and are particularly common in low income settings [6,17]. The prevalence of RTIs and STIs (sexually transmitted infection) in women (15–44 years old) in India increased by 26% and in Odisha by 126% between 1998–99 and 2002–04 (reported in the two rounds of District Level Household Survey—Reproductive and Child Health (DLHS-RCH)) [18]. Prevalence of RTI/STI in Odisha in the DLHS-RCH (2002–4) survey was 35.2% [18]. We are aware of very few population based prevalence surveys of bacterial vaginosis conducted in India [19], and none in Odisha. Surveillance studies on BV are mostly based on specialist clinic settings, such as genitourinary medicine clinics [20], gynaecology and antenatal clinics [21], which underestimate the true burden of disease in the community given the high proportion of asymptomatic or unreported cases [19].

Urinary tract infection (UTI) is the most common type of infectious disease in community practice after respiratory tract infection. A study conducted to determine the prevalence of community acquired-UTI in rural Odisha showed that prevalence of UTI in females was 45.2% [22]. Urinary tract infections are believed to be among the most common form of infection in girls and women of menstruating age and this is held to be due to unhygienic practices [23]. The exact biological mechanism by which, unhygienic MHM practices affect BV and UTI is not clear, but one possibility is that MHM creates abnormally moist conditions in the urogenital area that promotes opportunistic infection and imbalance in microbiota.

Most of the studies in the literature [24] which aim to investigate the association between menstrual management and health outcomes used RTI endpoints, but only a few of them employed clinically or laboratory confirmed bacterial vaginosis (BV) assays [24]. The remainder relied on self-reported vaginal discharge [24]. The only case-control study performed to explore MHM practices and health outcomes addressed secondary infertility [25], and only one study looked at urinary tract infection as another possible related outcome [26].

When trying to explore menstrual hygiene practices, all the papers found in the literature used self-reported methods [24]. Most of the studies compared mainly the types of absorbent used, e.g. rags vs. disposable pads, but a minority compared the methods of washing of cloths used for absorption or other menstrual hygiene practices [5,24,27].

We conducted the current study with the following primary aims:1) to assess the association between different menstruation hygiene management practices and the risk of symptomatic urogenital disease, 2) to examine the association between these practices with laboratory-confirmed infection (BV and UTI) and 3) to investigate the influence of other contextual factors, like sanitation and water access, and socioeconomic status on the association between different MHM practices and symptoms or laboratory-confirmed disease.

## Material and Methods

### Study sites

Between September 2013 and May 2014, a case-control study was conducted at Capital Hospital, Bhubaneswar, Odisha, and at Ispat General Hospital, Rourkela, Odisha. Capital Hospital, a Government of Odisha hospital with 647 beds, has all major specialities including Obstetrics and Gynaecology (O&G) and Family welfare. The hospital caters to the health care needs of roughly 1.2 million inhabitants of Bhubaneswar, including peri-urban areas and adjoining rural areas. Being a public funded institution, almost all services are available free of cost, hence, the majority of subjects attending this hospital come from financially disadvantaged groups. Ispat General Hospital (IGH), Rourkela, is situated in Sundergarh District of Odisha State. IGH is a 700 bed modern hospital managed by the Steel Authority of India Limited for free treatment of its employees and dependents. Those who are not eligible for free treatment can also attend the hospital with nominal payment. Since the hospital is situated in Sundergarh District, inhabited predominantly by a tribal population, a large proportion of the patients attending the hospital are from tribal populations. The participants were recruited from women who sought care from the gynaecology department for abnormal vaginal symptoms, and from women attending the Family welfare department for anti-fertility measures, specifically for intra-uterine devices (IUD).

### Selection of cases and controls

Symptomatic cases were non-pregnant women in the menstruating age (between 18–45 years) attending the gynaecology outpatient department (OPD) of Capital hospital and Ispat general hospital for complaints of one of the following 4 symptoms: abnormal vaginal discharge (unusual texture and colour, more abundant than normal), burning or itching in the genitalia, burning or itching when urinating, or having genital sores.

Asymptomatic controls consisted of women who lacked all of the above symptoms and who attended the O&G OPD of both hospitals for other complaints, (such as breast problems, irregular menstruation), as well as women attending family welfare OPD for intra uterine devices (IUD).

We excluded pregnant women, women who were currently menstruating, who had a hysterectomy, who had taken a course of antibiotics within the last three weeks, women with severe medical disorders requiring immediate referral to a higher level of health care and women refusing to give consent and donate a vaginal swab and a urine sample for analysis.

### Sample Collection

Informed written consent was obtained after the treating doctor identified the woman for recruitment to the study. A speculum examination of vagina and cervix was done by the O&G specialist, specifically to look for the presence of inflammatory changes. Simultaneously, vaginal specimens from posterior vaginal fornix were collected using three BD BBL swabs (BD, Maryland, USA). The first swab was used for vaginal pH measurement and for whiff test after addition of 10% KOH. The second swab was used for preparation of saline wet mount and smear for Gram staining and the third swab was stored for future studies. Midstream urine samples were also collected from participants for urine culture. Urinary samples were collected after swabs and questionnaire collection. After labelling, all materials (slides, urine container, and swabs) were transported immediately to the laboratory in a cooler with ice packs for microscopic wet mount examination, Gram staining and urine culture.

### Diagnostic assessment

Diagnosis of BV was done using Amsel’s clinical diagnostic criteria [12]. Bacterial vaginosis is considered positive if 3 of the following 4 criteria are met: (i) thin, whitish, homogeneous discharge (ii) a vaginal pH value greater than 4.5 (iii) a positive “whiff test” (“fishy” odour from KOH-treated wet-mount material) (iv) the presence of clue cells upon microscopic examination.

Urinary tract infections were diagnosed by culturing midstream urine samples in HiChrome UTI Agar (Himedia) plate. The plates were incubated under aerobic conditions at 37°C and read after 48 hours. A sample with at least 100,000 colony-forming units of a urinary pathogen (being *Escherichia coli* the most common, but also *Enterococcus faecalis*, *Staphylococcus aureus*, *Klebsiella species*) per milliliter in culture was considered positive [28].

### Outcomes of interest

**Fig 1:**
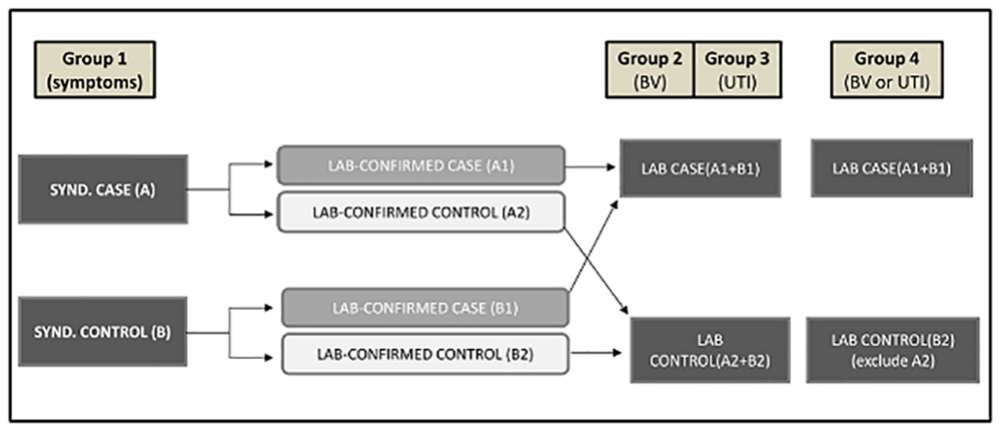
Definition of case-control outcomes groups.

Given the stigma associated with menstrual hygiene and sexual health in Indian society, we elected to use a clinic-based case-control study to overcome challenges in recruiting sufficient numbers of women admitting to having symptoms and women without symptoms willing to provide vaginal and urine samples. Based on the study design using symptomology and solicitation of health care services to identify case participants, our Group 1 analysis defined cases as women experiencing one or more symptoms of urogenital disease versus controls as those with no symptoms (Fig 1).

Based on the results of the laboratory BV or UTI tests, we created 2 additional groups of cases and controls to test for association between diagnostic tests for 2 common urogenital diseases hypothesized to be affected by poor menstrual hygiene.

Group 2: all participants whose laboratory tests were positive for BV regardless of symptoms (BV lab positive), versus those whose tests were negative for BV (BV lab negative).

Group 3: all participants whose laboratory tests were positive for UTI regardless of symptoms (UTI lab positive) versus those whose tests were negative for UTI (UTI lab negative).

Finally, our Group 4 accounts for the fact that some of the BV and UTI negative controls in Groups 2 and 3 could have had symptoms due to other related diseases. Cases were defined as those with a positive test result for either UTI or BV (BV or UTI lab positive). Controls were negative for both laboratory assays and excluded those with symptoms of other unknown lower urogenital infection (BV or UTI lab negative) (Fig 1).

### Risk factor data collection

After collection of the vaginal and urinary specimens, trained female interviewers collected information on socio-demographic and economic factors, clinical aspects and menstrual hygiene management (MHM) practices related with the absorbent material, the participant’s body hygiene habits and the water and sanitation condition in their households using a standardized questionnaire (S1 Fig).

#### Exploratory variables

The specific question used regarding type of absorbent was “What was the most common absorbent material you used during the last 6 cycles?” Further detailed information on the frequency and place for absorbent change during menstrual periods, body hygiene washing practices during menstruation (frequency, whether washing was done with soap and water) were also collected. Respondents who reused absorbents were also asked to describe absorbent hygienic practices: how absorbents were washed (including use of detergents), dried, packaged and stored. Respondents were also asked if they changed their MHM practices due to symptoms. The history of recurrence of the presenting symptoms was collected from symptomatic cases. Data were also collected on current use of contraceptives by sexually active respondents.

#### Confounding variables

Information on socio-demographic and economic characteristics, such as age, marital status, living arrangement, religion, caste, educational attainment, occupation, and wealth was also recorded. A wealth index was created by combining data on household possessions and housing characteristics using principal component analysis [29]. Data on toilet access (defined as having a toilet at home versus no toilet) and the main source of water (defined as having water at home versus having water outside the home (at a neighbour’s or relative’s house or in a public place) were also collected.

All the questionnaires were translated and administered in the Odia language.

### Sample size calculations

If we expect a 75% increase in odds of exposure in cases compared to controls, and the prevalence of unfavourable menstrual hygiene practices to be 30%, the minimum sample size of cases and controls, using an unmatched case-control design and 80% power at 5% level of significance testing was calculated as 240 in each group.

### Data handling and Statistical analysis

All data were double-entered into EpiData and analyzed using Stata 13.

Pearson χ^2^ tests were used for initial examination of associations between exposures and each outcome (1) symptomatic versus asymptomatic, 2) BV cases versus controls, 3) UTI cases versus controls, 4) ≥ 1 positive laboratory result for UTI or BV versus asymptomatic BV and UTI negative controls. To estimate the odds ratios and the associated 95% confidence intervals (CIs) for factors in relation to each outcome we used unconditional logistic regression. Potential determinants of symptoms or laboratory diagnosed disease were examined using a conceptual framework with 3 levels: sociodemographic characteristics, menstrual hygiene practices related with body hygiene, and household enabling environment (access to water and sanitation resources for practicing safe MHM). Final multivariable models were created through stepwise elimination of variables of interest from univariate analysis (P<0.1) while age, education and wealth index variables were retained in all the models. Owing to the collinearity between menstrual hygiene practices, the effect of each practice on the risk of symptoms or laboratory confirmed disease was evaluated in separate multivariable models adjusted for age, education and wealth index and by the other variables of interest from univariate model. Potential interacting variables were evaluated by including an interaction term in the regression model which was retained if statistically significant (P<0.05) by likelihood-ratio test.

### Ethical approval

The study was approved by the Institutional Review Board of Asian Institute of Public health (AIPH) and IGH, Rourkela, Odisha and by the Ethical Committee of Government of Odisha. The study was also approved by London School of Hygiene and Tropical Medicine (LSHTM ethics ref: 6520). Only those who provided written informed consent to participate in the study were included. Biological samples and questionnaires were labelled with a unique identifier to ensure confidentiality of participants.

## Results

**Fig 2:**
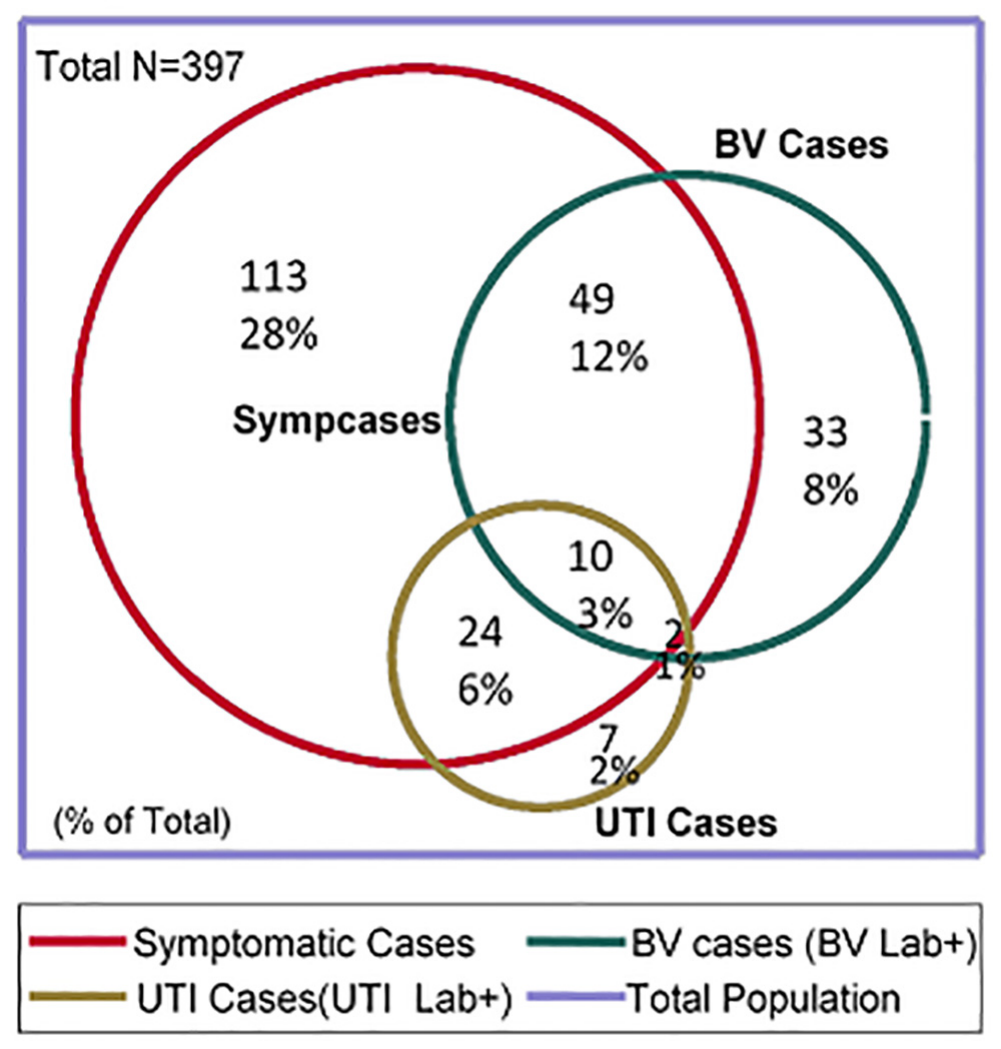
Venn diagram showing distribution of participants according to the different outcomes groups.

During the study a total of 486 women visiting the clinics were recruited (N = 247 in Bhubaneswar and N = 239 in Rourkela). 228 symptomatic cases and 258 asymptomatic controls were included in the study. All the participants provided swabs for testing BV but only 397 provided a urine sample for UTI testing. The urine had to be collected by women after the swabs and the questionnaire were collected, and in most cases the participants had to leave for other obligations and did not bring the samples back. The distribution of laboratory diagnosed BV and UTI among symptomatic cases is shown in the Venn diagram (Fig 2).

Table 1 shows the socio demographic characteristics of symptomatic cases and controls.

**Table 1:** Univariate analysis assessing association between demographics characteristic of women participating in the study according to symptomatic status.

	Cases no (%) (N = 228)	Control no (%) (N = 258)	OR*	95% CI*	p-value
**Average increasing age of participants (SD)**	31.2(7.1)	32.6 (7.7)	0.97	0.95–0.99	**0.04**
**Marital status**					
Single, never married	31 (13.6)	38 (14.8)	1.0		
Married	195 (85.5)	216 (84.2)	1.1	0.6–1.8	0.7
Widowed	2 (0.9)	4 (1)	na		
**Average Years Married**					
1 to 3 years	21 (10.5)	17 (7.8)	1.0		
4 to 12 years	95 (47.5)	81 (37.3)	0.9	0.5–1.9	0.80
13 to 32 years	84 (42.0)	119 (54.8)	0.6	0.3–1.1	0.10
**Religion**					
Hindu	202 (88.6)	230 (89.1)	1.0		
Muslim	6 (2.63)	5 (1.9)	1.4	0.4–4.5	0.6
Christian	14 (6.14)	19 (7.4)	0.8	0.4–1.7	0.6
Other	6 (2.6)	4 (1.5)	1.7	0.4–6.1	0.4
**Education level**					
Secondary or more	179 (78.5)	204 (79.1)	1.0		
Some primary	29 (12.7)	30 (11.6)	1.1	0.6–1.9	0.72
None	20 (8.7)	24 (9.3)	0.9	0.5–1.7	0.9
**Occupation**					
Employed or self-employed	32 (14.1)	40 (15.5)	1.0		
Housewife	167 (73.6)	188 (72.9)	1.1	0.6–1.8	0.6
Student	23 (10.13)	27 (10.5)	1.1	0.5–2.2	0.8
Other	5 (2.2)	3 (1.6)	2.1	0.4–9.3	0.3

*OR, odds ratio; CI, confidence interval, SD (standard deviation)

The average age of participant in this study was 32 (range 18–45). Cases were slightly younger than controls (31.2 vs. 32.6 years of age, p = 0.04). Otherwise, cases and controls did not differ significantly with respect to marital status, average years of being married, religion, education level, occupation, and wealth index. Most of the participants in the study were married, and only a small proportion of single women participated in the study (85% vs. 14%). Their average age at their first period was 13.4 (SD = 4.9). Most of the participants were Hindu (89%) and the primary occupation of the participants was housewife (73%). Thirty six percent of participants used condoms as a contraceptive method, followed by 32% who had a tubal ligation, 13% using an intrauterine device and 13% contraceptive pills or injections. Thirty-four percent of cases reported that the symptoms motivating attendance at the clinic were recurrent, but only 18 changed their MHM practices and only 11 changed the type of absorbent (using disposable ones when interviewed) as a result of symptoms. Among women who used reusable cloths, the odds of being a case were the same as among women who used disposable pads (OR = 0.7 95% CI 0.1–5.4, p = 0.7).

Univariate analysis showed that certain menstrual hygiene practices were associated with being a symptomatic case (Table 2). Women who used reusable cloths were 2x more likely to be a case than women using disposable absorbents (95% CI 1.4–2.9, p<0.001). Washing (bath or vaginal wash) with water only as compared with water and soap during menstruation was associated with symptomatic cases (95% CI 1.01–5.7, p = 0.045). Other practices such as number of absorbent material changes, staying at home when menstruating, the place where the absorbent material was changed and washing practices during menstruation (only vagina or body or both) did not differ significantly between cases and controls. Regarding the household enabling environment, we found that women whose water source was outside their home were 1.5x more likely to be a case compared with women who had the water source inside their home (95% CI 1.0–2.2 p = 0.06) but the association was weak. Cases and controls were similar with regard to sanitation access (having a latrine at home).

**Table 2:** Univariate analysis assessing association between different menstrual hygiene management practices and aspects of the household enabling environment for women according to symptomatic status.

	Case no (%) (N = 228)	Control no (%) (N = 258)	OR*	95% CI*	p-value
**Menstrual hygiene practices**					
**Absorbent material**					
Disposable pads	90 (39.5)	147 (57.0)	1		
Reusable cloths	138 (60.5)	111 (43.0)	2.03	1.4–2.9	**0.0001**
**Number of changes/day**					
3 times	75 (37.7)	100 (45.5)	1		
Twice	107 (53.7)	99 (45.0)	1.44	0.96–2.2	0.07
Once	17 (8.5)	21 (9.6)	1.07	0.5–2.2	0.83
**Stay home while menstruating**					
No	46 (20.2)	61 (23.6)	1		
Yes	182 (79.8)	197 (76.4)	1.22	0.8–1.9	0.4
**Place where absorbent is change**					
Outdoors	9 (3.9)	14 (5.5)	1		
At private room	63 (27.9)	54 (21.0)	1.81	0.7–4.5	0.2
At household toilet	154 (68.1)	189 (73.5)	1.26	0.5–3.0	0.6
**Washing practice during menstruation**					
Both (body and vagina)	189 (82.9)	223 (86.4)	1		
Only bath of full body	38 (16.7)	34 (13.2)	1.31	0.8–2.2	0.3
Only vaginal wash	1 (0.44)	1 (0.4)	1.17	0.07–18.9	0.9
**Frequency of washing during menstruation**					
Twice a day	50 (21.9)	67 (26.0)	1		
Once a day	173 (75.9)	190 (73.6)	1.22	0.8–1.9	0.4
Only first day	5 (2.2)	1 (0.4)	6.7	0.8–59.2	0.08
**Way of washing yourself during menstruation**					
water and soap	208 (91.2)	250 (96.9)	1		
water only	16 (7.02)	8 (3.1)	2.4	1.01–5.7	**0.048**
**Household Enabling Environment**					
**Access to a latrine**					
Yes	178 (78.1)	215 (83.7)	1		
No	50 (21.9)	42 (16.3)	1.43	0.9–2.3	0.12
**Improved Water source for ablution and bathing at/near household**					
In your own house	158 (69.3)	198 (76.4)	1		
Outside home	70 (30.7)	60 (23.3)	1.46	1.0–2.18	0.06
**Wealth index**					
Poorer	85 (37.4)	84 (33.1)	1.0		
Wealthier	142 (62.6)	170 (66.9)	0.82	0.6–1.2	0.3

*OR, odds ratio; CI, confidence interval, no = number. Denominators vary as not all respondents answered all questions

In order to explore if all these menstrual hygiene practices and the other exposure factors were also associated with specific infections—laboratory confirmed BV and UTI or with at least one positive test, we performed univariate analysis between the same exposure variables and the 3 different laboratory-confirmed outcomes (Table 3).

**Table 3:** Multivariable table with crude and adjusted OR for each model: Group 1: reported symptoms of urogenital disease or inflammation, Group 2: Bacteria vaginosis laboratory definition of cases and controls (BV lab confirmed), Group 3: Urinary tract infections laboratory definition of cases and controls (UTI lab confirmed), Group 4: BV or UTI cases laboratory confirmed and controls laboratory negative for both (excluding symptomatic controls) (BV/UTI lab confirmed).

					Clinical/Laboratory confirmed results											
	Group 1: Symptoms				Group 2: BV lab confirmed				Group 3: UTI lab confirmed				Group 4: BV/UTI lab confirmed			
	uOR	95% CI	Adj OR	95% CI	uOR	95% CI	Adj OR	95% CI	uOR	95% CI	Adj OR	95% CI	uOR	95% CI	Adj OR	95% CI
**Menstrual hygiene practices**																
**Absorbent material**																
Disposable pads	1		1		1		1		1		1		1		1	
Reusable cloths	2.03	1.4–2.9	2.26	1.5–3.4	1.5	1.0–2.3	1.23	0.8–2.0	1.95	1.01–3.8	2	1.0–4.0	3	1.9–4.5	2.8	1.7–4.5
**Number of changes/day**																
3 times	1		1		1				1				1		1	
Twice	1.44	1.0–2.2	1.4	0.9–2.2	1.5	0.9–2.5	1.4	0.8–2.3	1.6	0.8–3.5	1.5	0.7–3.3	1.5	0.9–2.3	1.2	0.7–1.9
Once	1.07	0.5–2.2	1.12	0.5–2.3	1.1	0.5–2.7	1.1	0.5–2.7	2.7	0.9–8.1	2.6	0.8–8.0	1.1	0.5–2.4	1.0	0.4–2.3
**Place where absorbent is change**																
Outdoors	1		1		1		1		1		1		1			
Indoors	0.88	0.6–1.2	0.97	0.6–1.5	0.58	0.4–0.8	0.56	0.3–0.9	0.6	0.4–1.1	0.5	0.3–1.1	0.6	0.4–0.9	0.9	0.5–1.6
**Frequency of washing**																
Twice a day	1				1				1				1		1	
Once a day	1.2	0.8–1.9	1.2	0.8–1.8	0.9	0.5–1.5	0.9	0.5–1.4	2.23	0.9–5.8	1.08	0.8–5.6	1.0	0.6–1.6	0.96	0.6–1.6
**Way of washing yourself during menstruation**																
water and soap	1		1		1				1				1		1	
water only	2.4	1.0–5.7	2.2	0.5–5.8	1.2	0.5–3.1	0.9	0.3–2.4	2.01	0.6–6.3	2.4	0.7–8	1.3	0.5–3.4	1.1	0.4–2.9
**Demographics and Income**																
**Average increasing age of participants**	0.97	0.9–1.0	0.97	0.9–1.1	1.01	0.9–1.0	1.01	0.9–1.1	0.99	0.9–1.0	0.99	0.9–1.0	0.97	0.9–1.0	0.98	0.9–1.01
**Education**																
Secondary or more	1		1		1		1		1		1		1.0		1.0	
Some primary	1.1	0.6–1.9	0.83	0.4–1.6	1.4	0.7–2.6	0.9	0.4–1.8	2.75	1.2–6.6	3.1	1.2–7.9	1.7	0.9–3.2	1.1	0.5–2.5
None	0.9	0.5–1.7	0.74	0.3–1.7	1.4	0.6–2.9	0.9	0.4–2.1	0.63	0.1–3.0	0.7	0.1–3.8	2	1.0–4.2	1.3	0.5–3.6
**Wealth index**																
Poorer	1		1		1		1		1				1.0		1	
Wealthier	0.82	0.6–1.2	1	0.6–1.8	0.6	0.4–0.9	0.5	0.3–0.9	0.9	0.4–1.7	1.5	0.6–3.9	0.44	0.3–0.7	0.6	0.3–1.1
**Household Enabling Environment**																
**Access to a latrine**																
No	1		1		1		1		1				1		1	
Yes	1.43	0.9–2.3	1.2	0.6–2.5	0.64	0.4–1.0	1.15	0.7–2.0	0.8	0.4–1.7	0.8	0.2–2.6	0.44	0.3–0.8	0.8	0.4–1.9
**Improve water source for ablution and bathing at/near household**																
In your own house	1		1		1		1		1		1		1		1	
Outside home	1.46	1.0–2.2	1.2	0.6–2.1	1.54	1.0–2.4	1.0	0.5–2.2	1.5	0.8–3.0	1.1	0.3–5.6	2.1	1.3–3.4	1.01	0.5–2.3

uOR, unadjusted Odds ratio, AdjOR, adjusted Odds ratio, CI, confidence interval.

Denominators vary as not all respondents answered all questions.

Unadjusted analysis showed that using all three outcomes, laboratory positive participants were more likely than laboratory negative patients to use reusable instead of disposable pads, and to change their absorbent outdoors vs. indoors (in a private room or a latrine). Regarding BV or UTI-specific disease, laboratory positive participants also changed their absorbent less often (weak association). UTI alone was weakly associated with less frequent washing during menstruation. Lower income, poor water access and lack of a latrine in the household were also associated with being a BV and a BV/UTI laboratory positive participant.

After multivariable analysis for each outcome, using reusable cloths remained strongly associated with being a symptomatic case (AdjOR = 2.3, 95%CI1.5–3.4, p<0.001) or having either BV or UTI disease (AdjOR = 2.8, 95%CI1.7–4.5, p<0.001), weakly associated with UTI disease status (AdjOR = 2.0, 95%CI1.0–4.0, p = 0.06), and not associated with BV disease status (AdjOR = 1.23, 95%CI0.8–2.0, p = 0.4). In the BV laboratory confirmed group, women who changed their absorbent pad indoors (AdjOR = 0.56 95%CI, 0.3–0.9, p = 0.02) and women who were relatively wealthy (AdjOR = 0.5, 95%CI0.3–0.9, p = 0.04) were less likely to have BV. In the UTI laboratory confirmed group, women having a primary school education or lower compared with secondary or higher education level faced 3x greater odds of having a UTI (AdjOR = 3.1, 95%CI1.2–7.9, p = 0.02).

Among the subgroup of participants (N = 249) using reusable pads, we explored whether different washing, drying and storing practices were associated with having BV or UTI or both diseases (Table 4). We found no significant associations between BV or UTI laboratory positive and negative participants for any of the washing, drying, and storing methods used.

**Table 4:** Univariate analysis among women who use reusable cloth pads (N = 249) comparing the odds of different MHM practices for washing, drying and storing cloth pads among BV or UTI laboratory confirmed cases and BV or UTI laboratory negative controls (excluding symptomatic controls) (Group 4).

	Group 4: BV/UTI lab confirmed				
	BV or UTI lab positive Cases no (%) (N = 138)	BV or UTI lab negative Control no (%) (N = 111)	OR	95% CI	p-value
**Washing place for sanitary cloth**					
Toilet	89 (69)	45 (78)	1.0		
Tube well or yard	18 (14)	5 (8.6)	1.8	0.6–5.2	0.3
Pond or river	23 (18)	8 (13.8)	1.6	0.6–3.5	0.4
**Way of washing sanitary cloth**					
Water and soap	125 (96.2)	54 (96.4)	1.0		
With water	5 (3.9)	2 (3.6)	2.3	0.2–5.7	0.9
**Drying method for sanitary cloth**					
In open space or sun	113 (87)	50 (89.3)	1.0		
Inside house	17 (13.1)	6 (10.7)	1.3	0.5–3.4	0.7
**Store place for sanitary cloth**					
Wrapped with polythene	116 (89)	52 (93)	1		
Wrapped with another material	7 (5.3)	3 (54)	1.04	0.3–4.2	0.9
Wrapped with nothing	8 (6.1)	1 (1.8)	3.6	0.4–29.4	0.2
**Placer where sanitary cloth is stored**					
In the toilet	24 (18.6)	15 (28.3)	1		
Hidden within cloths	17 (13.2)	1 (1.9)	10.6	1.3–88.3	0.03
In some place of the changing room	88 (68.2)	37 (69.8)	1.03	0.7–3.1	0.3

*OR, odds ratio; CI, confidence interval, no = number.

Denominators vary as not all respondents answered all questions.

## Discussion

This study provides support for the hypothesis that some menstrual hygiene practices can increase the risk of urogenital symptoms. Women who used reusable absorbent pads were more likely to have symptoms of urogenital disease than women using disposable pads. No other MHM practices were associated with symptoms after adjusting for confounders and other MHM practices. This association was also observed in another cross-sectional study in Indian women who reused cloth during menstruation and who self-reported vaginal discharge, but the authors did not adjust for any confounders [30].

When we assessed the association of the different MHM practices with specific disease outcomes, we observed that the effect of type of pad on BV disappeared when adjusted for other factors. These results are consistent with two studies which did not observe a significant association between clinically-confirmed BV (using Nugent criteria) and reuse of menstrual cloths [31,32]. However, two other studies, using clinically-confirmed BV as an outcome, did identify a significant association. Baisley et al [1] showed an association of adjOR = 1.42 (p = 0.02) using Nugent criteria for clinical confirmation in Tanzanian women, and Balamurugan [7] observed stronger effect (OR = 3.41) using Amsel criteria in Indian women, although the last study did not adjust for any confounders. Wealth and the place where a woman changes her pads during menstruation were the only factors associated with BV in our study after accounting for other contributing factors. The link between socioeconomic status and reproductive health has been established before [33], and it is plausible that increased wealth is associated with overall better hygiene resulting in lower susceptibility to BV and other infections. The findings from our study are unique in demonstrating that the type of pad is not the only important factor to consider, but that other factors related to having privacy and comfort for MHM are also important. Ignoring the minority of 23 women who changed outdoors, the effect of changing in a toilet was protective. However, none of these associations with access to WASH facilities was significant. A woman changing her menstrual absorbent outdoors is more prone to have BV than if she can change in a private place (a private room or toilet). This study cannot identify the causal source of this association, but it opens new questions about the relevance of women having a secure and comfortable place where they can change without stress and be closer to home with better proximity to water and other hygienic materials.

The effect of the type of pad on UTI also decreased after adjusting for other factors (p = 0.06) but was still relevant, as was the protective effect of higher education on UTI. With education, people are better prepared to prevent disease and to use health services effectively [33,34]. The only paper in the publically available literature which studied the association between disposable versus reused cloth pads using self-reported urinary tract infections did not find any association [26]. Our report is the first endeavour where laboratory diagnostic tests were used to assess UTI disease status.

We observed differences in the effect of the different MHM practices on the specific health outcomes. This could be due to selection bias, as the criteria used to select cases and controls included symptoms that were more related to BV and only 1 symptom very specific for urinary infections (feeling or burning or itching when urinating). Another possible source of bias is that BV could be present in many asymptomatic women. However, what is more likely is that poor MHM creates unhygienic vaginal conditions that universally promote opportunistic infection and inflammation from a broad spectrum of diseases (e.g. candidiasis, dermatitis) [1,6]. As noted above, the use of reusable pads was strongly associated with symptoms and with BV/UTI status after excluding symptomatic controls (Group 4). This effect was significantly weakened when case-control classification was based solely on diagnostic test results for BV or UTI. Participants with diagnostic-negative test results could still be symptomatic due to alternative disease etiology. Given the association between poor MHM practices and symptoms, the use of diagnostic confirmation only, without conjoined clinical interpretation, could misclassify true cases as controls. This misclassification probably skewed upwards the odds that a control would practice poor MHM, and so diluted the association between MHM exposures and disease status. To control for the effect of these other infections in our estimates of association of MHM practices with a laboratory diagnosed test result, we created a fourth outcome definition where cases included all the participants that were positive for BV and/or UTI, and the controls were diagnostic and symptom negative. The observations from that group were nearly identical to what we observed in the symptom-based case-control group, validating this hypothesis. These findings support the argument that using symptoms to define cases and controls of urogenital infections could be used in future studies aiming to measure urogenital health impact of menstrual hygiene practices. Therefore from the findings of our study, we could define a case as a participant who reports one of the following 4 symptoms: abnormal vaginal discharge (unusual texture and colour, more abundant than normal); burning or itching in the genitalia; burning or itching when urinating; or having genital sores. A control could be defined as not having any of the above mentioned symptoms. The only case-control study found in the literature, which explored the link between MH practices and health outcomes solely addressed secondary infertility [25]. However, several cross-sectional studies have used self-reported symptoms as a measure of health outcomes, with the most commonly used self- reported symptoms being abnormal vaginal discharge and itching in the genitalia [24]. In most of these studies the presence of the above symptoms was positively associated with the use of reusable absorbent pads [24].

As the type of absorbent used is an important factor associated with infection and symptoms, we explored the possibility that hygiene practices, such as the way the women wash, dry and store their reusable cloths, were risk factors for infection (Table 4). We did not observe that participants practising more thorough hygiene practices such as washing the cloth with soap and water in a private or enclosed place like their toilet, or drying pads in the open and storing them in a plastic bag to protect them from other sources of contamination were protective for infection. However, this estimation of the effect is likely underpowered due to an inadequate sample size, as the primary objective was not to assess these associations. Another possible explanation is that managing your pad hygienically will probably comprise a mix of these practices and not only one. But in this study we see that, reusing pads has stronger potential to have a negative impact on woman’s health. Studies across Africa, South East Asia, South America and the Middle East have showed that the use of reusable absorbents for managing menstruation and unhygienic practices to clean them are very common [24]. Across these studies these practices are found to be particularly acute in rural areas and amongst women and girls in lower socio-economic groups.

There are several limitations to our analysis. This is an observational study and so we cannot determine causality of the observed associations. Residual confounding may have remained when adjusting for self-reported risk factors, especially in relation to menstrual practices. We also did not adjust for other possible related factors, such as sexual practices or other infections (STIs and HIV). Another limitation is that we only measured two specific infections related with urogenital diseases, but other infections could be associated with MHM that we did not include in this study.

Our study did not show that access to a latrine was associated with less urogenital disease, but this may simply reflect the fact that the study turned out to be underpowered, as 215/257 (84%) of the control group had access to sanitation. Similarly, 198/258 (77%) of the control group had access to an improved water source. Detection of the impact of water supply on infection, and a more nuanced understanding of the conditions on which it depends, may require a larger study. Meanwhile, we consider that our study does provide indirect evidence of the protective benefits of in-house water supply in the association with frequency of washing. Since in-house water supply facilitates more frequent washing, it is likely to have a similar (protective) impact on infection.

Strengths in our work include a good sample size, the use of an expert microbiological laboratory with external quality controls to diagnose BV and UTI and the fact that all of the doctors and interviewers involved were females, which assured a relaxed environment to discuss a stigmatized topic.

Larger studies should be done to explore the influence on infections of factors related to different types of reusable pads, especially all the practices related with washing, drying and storing of the pads.

Given the association of reusing pads with different symptoms or even being diagnosed with BV or UTI, and the high prevalence of women using reusable pads in India, more research is needed to investigate factors that could help women to adopt more hygienic menstrual practices with comfort and privacy. Attention should be paid to other components related to MHM such as the role of water and sanitation in providing an adequate environment for women, which has been ignored in the context of MH for many years.

## Supporting Information

S1 Fig Questionnaire for exposure data collection. (DOC)

S2 Fig Case-control study data. (XLSX)
