# Supplementary material for: Menstrual Hygiene Practices, WASH Access and the Risk of Urogenital Infection in Women from Odisha, India
Source: PLoS One. 2015 Jun 30;10(6):e0130777. doi: 10.1371/journal.pone.0130777 (PMC4488331; doi:10.1371/journal.pone.0130777)
Supplement: S1 Fig — (DOC) [file pone.0130777.s001.doc]

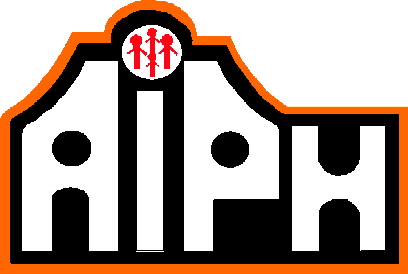
**ùidûe _eòù~ûR^û _ûAñ R^iõLýû ^òeúlY**

ÁWÿò ^´e

6

**i¹Zò _Zâ**

**_â{^^ ù~ûM Kùò**

**gûeú Gaõ ^ûeúcû^u EZiê**

**ûâ ae _eÃò**

**ûe _ezò**

**^ÜZû, _ePò**

**ûk^û Gaõ Gjû \ûß eû**

**_âbûaòZ ùjC[aô**

**û ù~û^ò I cìZûâ gdâ**

**_[ùe RúaûYê iõKcâ**

**Ye @baò**

**é¡ò @ûguû i´§ùe @^¤ê**

**û^ö**

**@õgMjâ**

**YKûeúu adi 18eê 45 ahð c¤ùe ùjûA[ôa I ùicûù^ _âR^^lc ùjûA[ôùa, ùijòcû^u _ûAñ Gjò i¹Zò _Zâ**

**aýajûe Keû~òaö ùicû^uê EZêiâûa icdùe _eòÃûe _eòz^ZÜ**

**_[ùe iõKâcYe @]ôK @ûguû i´§ùe _Peû~òaö**

**ûe _eòPûk^û Gaõ ù~û^òùe RúaûYê iõKcâ**

**Y Z[û cZê**

**âûgd**

**@û_Yuê gêùbzû ! ùcûe ^ûc ................................................................................... I cêñ IWògû eûRýe**

**bêaù^gße iÚòZ Giò@û^þ A^þÁòPêýUþ @`þ _aKäò**

**þ ùjf[þùe Kû~ðý Keê@Qòö EZêiâûa icdùe _eòÃûe _eòz^ÜZûe _ePò**

**ûk^û Gaõ**

**ù~û^ùò e RúaûYê iõKâcY Z[û cêZâûgd _[ùe @]Kô**

**iõKâcY bd, ~û@û @ûc ù\gùe iû]ûeY aýû]ô @ùUö ùi[ôù~ûMêñ @ûùc**

**MùahYû Keê@Qêö Gjò @¤d^ aòhdùe cêñ @û_Yuê KòQò KjQê**

**ò ö @û_Y Gjò MùahYûe @¤d^ùe @õg MâjY Keòùa Kò ^ûjó**

**@û_Y ^ò‰ðd Keùò a ö ^ò‰ðd ù^aû _ìaðeê, @û_Y ^òR _eòaûe Kò´û iµKðúdu Vûeê G aòhdùe _eûcgð ù^A_ûe«òö G[ôùe**

**RUòk g± c¤ [ûA_ûùe, ~ûjûe @[ð @û_Yuê aêSòaûùe ijR ùjûA^_ûùeö cêñ Kjê[ôaû icdùe aSê**

**òaûùe KQò**

**ò @iêaò]û ùjùf**

**cêñ Gjûe @[ð aSê**

**ò_ûeòfò ^ûjó ùaûfò ùcûùZ Kjòaûùe KòQò @iêaò]û ^ûjó, cêñ icd ù\A @û_Yuê bf bûaùe aêSûAù\aòö**

@û_Y _ùe @û_Yu cZ _eòað^ Keò _ûeòùa Gaõ @õg MâjYeê c¤ Ijeò ~ûA_ûeòùa, ~\ò aû _aì

ðeê eûRò

ùjûAQ«òö ~\ò @û_Y G[ôùe @õg MâjY ^Ke«ò, Zûùjùf @û_Yuê Gaõ @û_Yu _òfûKê Wÿûqeú PòKò›ûùe ùKøYiò _eòað^

**ùja ^ûjóö Gaõ _ìaðbkò icÉ PòKò›ûe iêaò]û _ûA_ûeòùaö**

**Gjò @¤d^ _âR^^ ù~ûMý Kùò gûeú Gaõ ^ûeúcû^u EZêiâûae _eòÃûe _eòz^ÜZû I _eòPûk^û, Gaõ ù~û^òùe**

**RúaûYê iõKâcY Z[û cêZâûgd _[ùe iõKâcYe @]ôK @ûguû C_ùe Keû~ûCQòö @ûù¸cûù^ @û_Yuê EZêiâûae _eòÃûe**

**_eòz^ÜZû I Gjûe _eòPûk^û aòhdùe KQò**

**ò _âgÜ _Pûeòaêö iõKâcòZ ùjûA[ôùf, EZêiâûa ùaùk aýajûe ùjûA[ôaû K_Wÿû /**

**_ýûW,ÿ**

**aûjûeê[ôaû Zek _\û[ð I _eiò**

**âûe ^cê^û @û_Yu Vûeê iõMâj Keòaêö cìZâ ^kû I ù~û^òùe iõKâcYKûeú RúaûYê ejòQ«ò**

**Kò ^ûjó @ûù¸ _eúlû Keò ù\Lôaêö Gjò MùahYû _ûAñ @û_Yu Vûeê iõMéjúZ icÉ Z[ý @ûù¸ ùMû_^ eLôaêö**

Gjò MùahYû \ßûeû @û_Yue ùKøYiò lZò _jôaûe i¸ûa^û ^ûjó, ù~ùjZê @ûù¸ ùKak EZêiûâ a ùaùk aýajûe

**ùjûA[ôaû K_Wÿû / _ýûWÿ, aûjûeê[ôaû Zek _\û[ð I _eòiâûe ^cê^û @û_Yu Vûeê iõMâj aýZúZ @ûù¸ @û_Yu geúe bòZeKê**

**ùKøYiò Rò^òh _ùâ ag KeûA ^cê^û iõMjâ**

**Keò _eúlû Kò´û @^ý ùKøYiò ^ìZ^ _eúlû Keòaê ^ûjóö @û_Y _ûC[ôaû PKò**

**ò›ûùe**

ùKøYiò _eòað^ Keû~òa ^ûjóö ~\ò @û_Y Gjò @¤d^ùe @õg MâjY KeòaûKê Pûjñû«ò ùeûMe ^ò\û^ _ûAñ KeòaûKê [aô

û _eúlû

**MêWKÿò**

**ê @û_Yuê aò^û Lþyùð e còkò_ûeòaö Gjû aýZúZ @û_Yuê @^ý ùKøYiò iêa]ò**

**û còkò ^_ûùe, Kò«ê @û_Y Gjò @^ê¤û^ùe ù~ûM**

**ù\aû \ßûeû Gjò ùeûMe KûeY RûYòaûùe @û¸uê ijûdK ùjaö ~\òI icûRKê MùahYû Pûfê[ôaû icdùe ùicòZò KòQò iaê**

**còkò^_ûùe, Kò«ê baòhýZùe Gjûe `kû`k ^ìZ^ PòKò›û _¡Zòùe ijûdK ùjûA_ûeòaö**

**ò]û**

**~\ò G[ôùe @õg MâjY KeòaûKê Pûjñû«,ò**

**@ûù¸ ù~Cñ ^cì^û MêWòK @û_Yu Vûeê iõMâj Keòaê, Zûjû Gi@ò**

**û^þ A^þÁPò**

**êýUþ**

@`þ _aäòK ùjf[eþ

Zßûa]û^ùe, iêelZò

bûùa Zûfû _KûA eLû~òaö @^ê¤û^ ieòMfû _ùe ~\ò KòQò ^cê^û akò_ùWÿ, Zûjûe

**baòhýZùe @]ôK _eúlû _ûAñ, @û_Y @^êcZò ù\ùf ZûjûKê iêelòZ eLôaêö Gjò @^ê¤û^ _ûAñ iõMéjZò**

`kû`kKê @ûù¸ ùa÷mû^òK i¹òk^úùe C_iÚû_^ Keò_ûeê Kò´û aòmû^bòòK _Zâ_ZâòKûùe Qû_ò_ûeê c¤ K«ò

**_eòPd ùKøYiò[ôùe _âKûgòZ ùja ^ûjó ö**

**Z[ý I iìP^ûe**

ê @û_Yue ^ûc I

**Gjò MùahYg _ûAñ _âÉûaUò G[Kô**

**ûf KcUò**

**ò \ßûeû @^êùcû\Zò**

**ùjûAQòö ù~Ccñ**

**ûù^ G[ôùe @õg MâjY Keòùa, ùicû^ue**

ù~_eò ùKøYiò lZò ^_jù Gjò KcòUòe cêLý CùŸgýö

**@ûù¸ @ûgû KeêQê ù~, @û_Y G[ôùe @õg MâjY Kùf, @û_Yue KòQò lZò ùja ^ûjóö ~\ò @û_Yu c^ùe KòQò _âgÜ**

**Kò´û Ròmiû @Q,ò**

**aêSò _ûeòùaö**

**Zûùjùf @û_Y - W.ÿ**

**_\àûkdû \ûi, G.@ûA._ò.GP.þ ,ù`û^ - 0674-6574656u ijòZ ù~ûMûù~ûM Keò**

**_éÂû 2e 1**

**1**

**_â{^^ ù~ûM Kòùgûeú Gaõ ^ûeúcû^u EZêiâûae _eòÃûe _eòz^ZÜ**

**û, _eòPûk^û Gaõ Gjû \ûß eû**

**_âbûaòZ ùjC[ôaû ù~û^ò I cZì**

**ûâ gdâ**

**_[ùe RúaûYê iõKcâ**

**ùNûhYû _Zâ**

**Ye @bòa¡é**

**ò @ûguû i´§ùe @^ê¤û^ö**

MùahYûe CùŸgý, _âYûkú, @^iê

eY _¡Zò, @ûguû I fûb i´§ùe ùcûùZ Kêjû~ûAQòö @^ê¤û^ _ûAñ ~ûjû iaê _âgÜ ejòQò

ùcûùZ _Pûeòaû _ûAñ cñê @^êcZò ù\AQò I cêñ aêSò_ûeê^[ôaû _âgÜe Ce ùcûùZ bf bûùa aêSû~ûAQòö ~\ò ùcû c^ùe KòQò _âgÜ

[ûG, @iêaò]û [ûG Kò´û MùahYû _ûAñ ^òRÊ cZûcZ Kò´û ùcûùZ còkò[ôaû iPì

^û _ûAñ cêñ Kûjû ijòZ ù~ûMûù~ûM Keòaò ùcûùZ

Kêjû~ûAQöò

i¹Zò _Zâ cêñ _XÿòQò / ùcûùZ _Xÿò gêYû~ûAQò Gaõ cêñ aêSòQò ù~, G[ôùe bûM ù^A ù~ùKøYiò icdùe @õg MâjYeê

Ijeò ~ûA_ûeòaòö ùcûùZ Kêjû~ûAQò ù~ \ÉLZ I ZûeòL [aô

**1. @û_Y G[ôùe @õgMâjY KeòaûKê AzêK Kò ?**

û i¹Zò _Zeâ

GKKZò

û ^Kf ùcûùZ \ò@û~òaö

(0) ^ûñ (_âgKÜ

ðû! Ce\ûZûuê ]^ýaû\ \ò@«öê

iûlûZKûe a¦ Ke«êö)

(1) jñ (_âgÜKðû : ^òcÜ _âgÜKê ~û@û«êö)

**2. @û_Yu Vûeê iõMjâ**

**òZ ^cì^û (ù~û^òeê Zek _\û[ð Gaõ aýajéZ K_Wÿû/_ýûWÿ) ~û@û MùahYû _ûAñ ù\AQ«ò ~\ò**

**ùi[ôeê KQò**

**\ò@«êö)**

**ò akò _Wÿòfû, Zûjû baòhýZùe _eúlû ^òeúlû Keaû _ûAñ @^êcZò ù\ùa Kò?(ùMûUòKùe UòKþ PòjÜ**

(0) Gjò ^cê^û (ù~û^eò

ê Zek _\û[ð Gaõ aýajéZ K_Wûÿ / _ýûWÿ) iõùM iõùM ^Á Keò ù\aûKê cêñ AzêK (_âgÜ

Kûð

: _eaðú _âgÜKê ~û@û«êö)

(1) Gjò ^cê^û (ù~û^òeê Zek _\û[ð I aýajéZ K_Wÿû / _ýûWÿ) @^ò¡ðÁò

Kûk _ûAñ iûAZò eLôaûKê @^cê Zò

ù\C@Qò (_âgÜ_ðû : ]^ýaû\ RYû«,ê

\ÉLZ ùfLû[ôaû iÚû^Kê ~û@û«êö)

**3. ~\ò ùcû ^cê^ûKê (ù~û^òeê Zek _\û[ð I aýajéZ K_Wûÿ eLôaûKê @^êcZò ù\aò-**

**/ _ýûWÿ) baòhýZùe _eúlû ^eò**

**úlû Keòaû _ûAñ**

(0) ^cê^ûeê ùcûe _ePò

dKê fòbûA ù\aûKê PûjQêñ òö

(1) ^cê^ûùe ùcûe _ePò

dKê eLôaûKê PûjQêñ òö

@õgMâjYKûeúu ^ûc :

@õg MâjYKûeúu \ÉLZ : ZûeLò (\^ò

~\ò @gòlòZ @U«ò :

/ cûi / ahð)

@õg MâjYKûeúuê i¹Zò _Zâ aòhdùe aSê

ûA \@ò

û~ûAQòö, cñê iûlú @ùU Gaõ Ce\ûZûuê _âgÜ _Pûeaò

ûe iùê

~ûM \ò@û~ûAQòö

cêñ ^ò½òZ ù~ @õg MâjUKûeú Zûue i¹Zò ÊAzûùe ù\AQ«òö

iûlýKûeúu ^ûc :

iûlýKûeúu \ÉLZ :

ZûeòL (\^ò

/cûi/ahð) :

@õgMâjYKûeúu Uò_ PòjÜ

i¹Zò ù^aû icdùe G.@ûA._ò.GPþ. iê_e bûAReue ^ûc :

G.@ûA._ò.GPþ. iê_ebûAReue \ÉLZ : ZûeLò (\^ò

/ cûi / ahð)

**Gjò i¹Zò_Zâe GKKòZû ^Kf @õg MâjYKûeúuê \ò@û~aò ö**

Participant ID Location: Date:

Participant ID will consist in a letter (B=Bhubaneswar participants, R=Rourkela participants), followed by 3 digits. Location, will refer to the area (city) or village where the participant come from.

**I. DEMOGRAPHIC INFORMATION**

RESPON DENT INFOR MATION (Fe m al e betwe en 14 and 45 years o f age)

**No. Question Answer Skip**

1. How old are you ?

2. What is your marital status (Select one)

3. Have you given birth within the last three months?

4. How old were you when you had your first period?

5. What is your religion?

6. What your caste or tribe?

...............................................................Years Old

**Yes No** a. Single, never married ........................ 1 .......... 2 b. Married ......................................... 1 .......... 2

i. How many years have you

been married ? .............................. 1 .......... 2 c. Widowed ......................................... 1 .......... 2 d. Divorced ......................................... 1 .......... 2 e. Separated ........................................ 1 .......... 2 f. Others ......................................... 1 .......... 2

**Yes No**

**........................................................ 1 .......... 2**

**................................................. Years Old**

**Yes No**

a. Hindu ......................................... 1 .......... 2

b. Muslim ......................................... 1 .......... 2 🡪 **7** c. Christian ......................................... 1 .......... 2 🡪 **7** d. Other ......................................... 1 .......... 2 🡪 **7**

**Yes No** a. Scheduled caste (SC) ....................... 1 .......... 2 b. Scheduled tribe (ST) ......................... 1 .......... 2 c. Other backward caste (OBC) ............ 1 .......... 2 d. Other caste ...................................... 1 .......... 2

**No. Question Answer Skip**

7. W hat is the highest standard year of education that you have completed ?

8. What is your occupation ?

**Yes No** a. No formal education ......................... 1 .......... 2 b. Some primary (1-4th year) ................ 1 .......... 2 c. Completed primary (5th year) ........... 1 .......... 2 d. Some secondary (6-10th year) .......... 1 .......... 2 e. Completed +2 year (12th year) .......... 1 .......... 2 f. Completed +3 year (university, etc) ... 1 .......... 2

**Yes No** a. Employed or self-employed ............... 1 .......... 2 b. Housewife ........................................ 1 .......... 2 c. Student ........................................ 1 .......... 2 d. Other ........................................ 1 .......... 2

**B. HOUSEHOLD ECONOMIC INFORMATION**

9. W here does cooking nor m all y take place? (Select one)

10. How many people live in your household?

11. How many rooms in the house are used for sleeping?

12.. What is the principal source of lighting for your household? (Select one)

**Yes No** a. Inside house, separate room ............ 1 .......... 2 b. Inside house, no separate room ....... 1 .......... 2 c. In separate building .......................... 1 .......... 2 d. Outdoors ........................................ 1 .......... 2 e. Other ........................................ 1 .......... 2

**........................................................ ................**

**........................................................ ..................**

**Yes No** a. Electricity ........................................ 1 .......... 2 b. Kerosene ........................................ 1 .......... 2 c. Gas ........................................ 1 .......... 2 d. Oil ........................................ 1 .......... 2 e. Wood or charcoal. ............................ 1 .......... 2 f. None ........................................ 1 .......... 2 g. Other ........................................ 1 .......... 2

**No. Question Answer Skip**

13. Does any usual member of this household have a bank account or a post office account?

14. Which is the monthly household income

(in rupees of all family members)?

15. Do any members of your household own any of the following items? (Select all that apply)

16. Does your household have any of the following? (Select all that apply)

17. Does your household own a BPL card? (please verify)

**Yes No**

**........................................................ 1 .......... 2**

**Yes No** a. Bellow 5,000 .................................... 1 .......... 2 b. 5,000-10,000 .................................... 1 .......... 2 c. 10,000-20,000 .................................. 1 .......... 2 d. 20,000-30,000 .................................. 1 .......... 2 e. 30,000 and above ............................ 1 .......... 2 f. Don’t Know ....................................... 1 .......... 2

**Yes No** a. Watch or clock ................................. 1 .......... 2 b. Pressure cooker ............................... 1 .......... 2 c. Telephone/Mobile Phone .................. 1 .......... 2 d. Television ........................................ 1 .......... 2 e. Refrigerator ...................................... 1 .......... 2 f. Radio ........................................ 1 .......... 2 g. Chair ........................................ 1 .......... 2 h. Mattress ........................................ 1 .......... 2 i. Cot or bed ........................................ 1 .......... 2 j. Table ........................................ 1 .......... 2 k. Electric fan ....................................... 1 .......... 2 l. Sewing machine ............................... 1 .......... 2 m. Water pump ..................................... 1 .......... 2 n. Bicycle ........................................ 1 .......... 2 o. Motorbike/Scooter ............................ 1 .......... 2 p. Car ........................................ 1 .......... 2 q. Animal drawn cart ............................ 1 .......... 2 r. Thresher ........................................ 1 .......... 2 s. Tractor ........................................ 1 .......... 2 t. Agricultural land ............................... 1 .......... 2

**Yes No** a. Cattle ........................................ 1 .......... 2 b. Buffalo ........................................ 1 .......... 2 c. Goats ........................................ 1 .......... 2 d. Sheep ........................................ 1 .......... 2 e. Chicken ........................................ 1 .......... 2 f. Pigs *............................................* 1 .......... 2

**Yes No** a. No ........................................ 1 .......... 2 b. Yes, verified ..................................... 1 .......... 2 c. Yes, not verified ................................ 1 .......... 2

**No. Question Answer Skip**

**2. Clinical Information**

**Lower reproductive symptoms:**

18. Do y ou hav e abno r m al v agi nal di s c har g e? ( m or e abund ant t han normally)

19. Do you have a feeling of burning or itching in the genitalia (v ulvovaginal area) ?

20. Do you have a feeling of burning or itching when urinating ?

21. Do you present genital sores ?

**Other symptoms:**

22. Do you have pain in your lower back ?

23. Do you hav e pain in y our bell y or stomach ?

24. Do you have pain in your breast ?

25. Hav e you had hysterectomy before ?

26. Do y ou p r esen t abn or m al v agi nal bleeding out of the menstruation days?

**Yes No**

**........................................................ 1 .......... 2**

**Yes No**

**........................................................ 1 .......... 2**

**Yes No**

**........................................................ 1 .......... 2**

**Yes No**

**........................................................ 1 .......... 2**

**Yes No**

**........................................................ 1 .......... 2**

**Yes No**

**........................................................ 1 .......... 2**

**Yes No**

**........................................................ 1 .......... 2**

**Yes No**

**........................................................ 1 .......... 2**

**Yes No**

**........................................................ 1 .......... 2**

**No. Question Answer Skip**

**Other related health questions:**

27. When was the last time that you had your period?

(write the date day/month/year

/ / )

28. Do you use any type of Contraceptive method at the moment?

29. If you use a contraceptiv e m ethod, choose which one?

30. Hav e you taken any antibiotic treatment during the last 2 weeks ?

31. Is the problem why you are coming to the clinic a recurrent one (it is happening more than once) ?

(If the answer is No, go to 35)

32. How often these problems appeared in the last year approximately ?

33. Did you change your menstrual hygienic habits after having this recurrent symptom?

34. If yes, what did you change?(mark with a x, the applied ones)

**Yes No** a. Yes ......................................... 1 .......... 2 b. No ......................................... 1 .......... 2 c. I don’t know ...................................... 1 .......... 2

**Yes No** a) Condom use .................................... 1 .......... 2 b) Injections or birth control pills ........... 1 .......... 2 c) Intrauterine device ............................ 1 .......... 2 d) Tubal ligation .................................... 1 .......... 2 e) Other ......................................... 1 .......... 2

**Yes No** a. Yes ......................................... 1 .......... 2 b. No ......................................... 1 .......... 2 c. I don’t know ...................................... 1 .......... 2

**Yes No**

a. Yes ......................................... 1 .......... 2

b. No .................................................... 1 .......... 2 🡪 **35**

**Yes No** a) Every Months ................................... 1 .......... 2 b) 6 times in a year ............................... 1 .......... 2 c) 4 times in a year ............................... 1 .......... 2 d) 3 times in a year ............................... 1 .......... 2 e) Other ......................................... 1 .......... 2

**Yes No**

**........................................................ 1 .......... 2** 🡪 **35**

**Yes No** a) Menstrual absorbent type ................. 1 .......... 2 b) Vaginal washing practices ................ 1 .......... 2 c) Body washing practices .................... 1 .......... 2 d) Absorbent washing practices (if applied)1 ....... 2 e) Place for changing menstrual absorbent1 ....... 2 f) Place to defecate or urinate every day 1 ........ 2 g) Place to defecate or urinate during

menstruation .................................... 1 .......... 2

**No. Question Answer Skip**

**3. Exposure information**

35. What was the most commonly absorbent material used during the last 6 cycles?

36. How often do you change the absorbent material on your heav iest day?

37. Do you normally stay at home when menstruating? yes/no

38. Where do you change your absorbent material when at home? (select the main one)

39. W hat do you do with the absorbent material? (select the main one)

**Yes No** a) disposable sanitary pads .................. 1 .......... 2 b) reusable cloths/towel ........................ 1 .......... 2 c) tampon ......................................... 1 .......... 2 d) nothing ......................................... 1 .......... 2 e) other: ......................................... 1 .......... 2

**Yes No** a) once a day ....................................... 1 .......... 2 b) twice a day ....................................... 1 .......... 2 c) Three times a day ............................ 1 .......... 2 d) other: ......................................... 1 .......... 2

**Yes No**

**........................................................ 1 .......... 2**

**Yes No** a) In a household toilet ......................... 1 .......... 2 b) In a private room in the house .......... 1 .......... 2 c) In a neighbors/relatives/public .......... 1 .......... 2 facility outside the house or yard ....... 1 .......... 2

d) In the bush/field/ground/river/

streamsite ........................................ 1 .......... 2 e) Other ......................................... 1 .......... 2 a. Specify ......................................... 1 .......... 2

**Yes No**

a) Dispose it (go to 43) ......................... 1 .......... 2 🡪 **45**

b) Reuse it ......................................... 1 .......... 2

**No. Question Answer Skip**

40. If you r euse it, where do you wash

it?(select the main one)

41. How do y ou wash y our s ani t ar y cloths?(select the main one)

42. After washing it, how do you dry the cloth?(select the main one)

43. How do you store the cloth for use next time?(select the main one)

**Yes No**

a) Inside the toilet stall. ......................... 1 .......... 2 b) In the public pond where people

normally have bath ........................... 1 .......... 2 c) In the pubic pond where people

dispose of trash ................................ 1 .......... 2

d) At the tube well ................................. 1 .......... 2 e) In the river ........................................ 1 .......... 2

f) I don’t wash it. .................................. 1 .......... 2 🡪 **43**

g) Other: ......................................... 1 .......... 2 (a) Specify ...................................... 1 .......... 2

**Yes No** a) With water ........................................ 1 .......... 2 b) With water and soap or detergent ..... 1 .......... 2 c) With water and mud/ash ................... 1 .......... 2 d) Other: ......................................... 1 .......... 2 (a) Specify ...................................... 1 .......... 2

**Yes No** a) Dry it in the sun or open space. ........ 1 .......... 2 b) Dry it inside the house. ..................... 1 .......... 2 c) I don’t dry it. ..................................... 1 .......... 2 d) Other: ......................................... 1 .......... 2 (a) Specify ...................................... 1 .......... 2

**Yes No** a) Wrapped in polythene ...................... 1 .......... 2 b) Wrapped in another material - .......... 1 .......... 2 c) Wrapped in nothing .......................... 1 .......... 2 d) Other: ......................................... 1 .......... 2 (a) Specify ...................................... 1 .......... 2

**No. Question Answer Skip**

44. Where do you store the cloth for use next

time?(select the main one)

45. If you will not reuse it, where do you dispose it? (select the main one)

**WASH questions:**

46. What type of washing (bath or vaginal wash) do you practice during(select the main one):

47. How often do you wash yourself (bath or v aginal wash) during(select the main one):

**Yes No**

a) Within my clothes ............................. 1 .......... 2 b) In the toilet ....................................... 1 .......... 2 c) In some place of the changing room. 1 .......... 2

d) Other: ......................................... 1 .......... 2

(a) Specify ...................................... 1 .......... 2

**Yes No** a) Inside latrine ..................................... 1 .......... 2 b) In a rubbish bin inside or close to

the latrine ......................................... 1 .......... 2 c) In the household rubbish bin. ............ 1 .......... 2 d) Put it in the pond .............................. 1 .......... 2 e) I burn it ......................................... 1 .......... 2 f) Discard in any other open space. ..... 1 .......... 2 g) Other: ......................................... 1 .......... 2

(a) Specify ...................................... 1 .......... 2

44.a 44.b

Menstruation ? Normal day?

a) Only vaginal wash. 01 01 b) Bath of full body. 02 02 c) Both 03 03 d) I don’t wash myself 04 04

45.a 45.b

Menstruation ? Normal day?

a) Once a day 01 01 b) Twice a day 02 02 c) Only the first day 03 03 d) Other: 04 04

(a) Specify......................................................

**No. Question Answer Skip**

48. What do you use to wash yourself (bath or vaginal wash) during(select the main one):

49. W here does your household normally obtain drinking water? Are there any other sources that you use? (Record primary and all reported sources)

50. W here i s the pr im ar y water sour ce located? (select one)

47.a 47.b

Menstruation ? Normal day?

a) Water only 01 01 b) Water and Soap or

Detergent 02 02 c) Water and Ashes 03 03 d) Water and soil/dirt/mud 04 04

Primary Anay

(Select One) (Select all)

a. Piped tap 01 01 b. Tube well or borehole 02 02 c. Protected well 03 03 d. Unprotected well 04 04 e. Protected spring 05 05 f. Unprotected spring 06 06 g. Rainwater 07 07 h. Tanker truck 08 08 i. Cart with small tank 09 09 j. Surface water (river,

dam, lake, pond, or stream, canal, irrigation

channel) 10 10 k. Bottled water 11 11 l. Other 12 12

**Yes No** a. In the house ..................................... 1 .......... 2 b. In the yard ........................................ 1 .......... 2 c. At a relativ e’s house or yard ............. 1 .......... 2 d. At a neighbor’s house or yard ........... 1 .......... 2 e. At a public location ........................... 1 .......... 2

**No. Question Answer Skip**

51. Where do you defecate more often during

(select the main one):

52. Where do you urinate more often during

(select the main one):

53. How many minutes does it take for you to get to your primary defecation site?

54. How far is the water source you use when going to defecation?

49.a 49.b

Menstruation ? Normal day?

a) Facility in house or yard 01 01 b) Facility in relative or

Neighbor’s house or yard 02 02 c) Facility in community 03 03 d) No facility-go in

household and Dispose of in bush/field/ground/

River/stream 04 04 e) No facility-go the bush/

field/Ground/river/stream 05 05

50.a 50.b

Menstruation ? Normal day?

a) Facility in house or yard 01 01 b) Facility in relative or

Neighbor’s house or yard 02 02 c) Facility in community 03 03 d) No facility-go in

household and Dispose of in bush/field/ground/

River/stream 04 04 e) No facility-go the bush/

field/Ground/river/stream 05 05

53.a When menstruation.............................min

53.b in a normal day......................................min

54.a When menstruation.............................min

54.b in a normal day......................................min

**No. Question Answer Skip**

55. Do you need to carry extra water when you go for urination when menstruating?

56. Do you need to carry extra water when you go for defecation when menstruating?

57. Is there a toilet facility in your household?

58. How long have you had access to a latrine at your household?

59. When do you use more the latrine during

(select the main one):

60. If you are menstruating, do you use the latrine (mark all the possible options)?(if the participant don’t have latrine skip to )

61. Do you use the latrine more often if you are menstruating?

62. W hen you are m enstruating, do you deliberately get up earlier in the morning in order to use the latrine earlier than you normal do?

**Yes No**

**........................................................ 1 .......... 2**

**Yes No**

**........................................................ 1 .......... 2**

**Yes No**

a) Yes ......................................... 1 .......... 2

b) No ......................................... 1 .......... 2 🡪 **69**

..........................................................................Years

57.a 57.b

Menstruation ? Normal day?

a) In rainy season 01 01 b) In dry season 02 02 c) The same in both seasons 03 03 d) I don’ t use the latrine 04 04

**Yes No** a) For changing menstrual absorbent. .. 1 .......... 2 b) For urinating ..................................... 1 .......... 2 c) For defecating .................................. 1 .......... 2 d) For washing my absorbent material. . 1 .......... 2 e) Other ......................................... 1 .......... 2

**Yes No**

**........................................................ 1 .......... 2**

**Yes No**

**........................................................ 1 .......... 2**

**No. Question Answer Skip**

63. Do you find enough privacy in the latrine you use?

64. Does your latrine have a roof ?

65. Does your latrine have a door ?

66. Do you hav e a hand-washing facility inside/or close to your latrine ?

67. Do you have a disposal place (bucket, container or pit) inside /or close to your latrine?

68. Where do you get your water for latrine use?(select the main one)

69. How do you normally wash your hands? (select the main one)

**Yes No**

**........................................................ 1 .......... 2**

**Yes No**

**........................................................ 1 .......... 2**

**Yes No**

**........................................................ 1 .......... 2**

**Yes No**

**........................................................ 1 .......... 2**

**Yes No**

**........................................................ 1 .......... 2**

**Yes No** a) There is a tap inside the latrine ......... 1 .......... 2 b) I bring it from my priv ate tube well .... 1 .......... 2 c) I bring it from a neighbor tube well

within 1 min of my home ................... 1 .......... 2 d) I bring it from a public source

within 1 min m of my home ............... 1 .......... 2 e) I bring it from a far source

(more than 5min walk) ...................... 1 .......... 2

**Yes No**

a) Water Only ....................................... 1 .......... 2 b) Water and soap or detergent ............ 1 .......... 2 c) Water and ashes .............................. 1 .......... 2 d) Water and soil/dirt/mud .................... 1 .......... 2

**No. Question Answer Skip**

70. When do you usually wash your hands? (Select all that apply. Prompt for any times they can think of, but do not probe by asking if they wash their hands at a specific time)

71. How do y ou wash your hands aft er defecation or menstruation?(select the main one)

**Yes No**

a) Before eating .................................... 1 .......... 2 b) After eating ....................................... 1 .......... 2 c) Before cooking ................................. 1 .......... 2 d) After handling domestic animals ....... 1 .......... 2 e) After defecating ................................ 1 .......... 2 f) After urinating ................................... 1 .......... 2 g) After cleaning child that defecated .... 1 .......... 2 h) After changing menstrual hygiene pad ... 1 .......... 2 i) Never ......................................... 1 .......... 2 j) Other ......................................... 1 .......... 2

**Yes No**

a) Water Only ....................................... 1 .......... 2 b) Water and soap or detergent ............ 1 .......... 2 c) Water and ashes .............................. 1 .......... 2 d) Water and soil/dirt/mud .................... 1 .......... 2 e) I don’t wash my hands ...................... 1 .......... 2
